# Lessons from national biobank projects utilizing whole-genome sequencing for population-scale genomics

**DOI:** 10.1186/s44342-025-00040-9

**Published:** 2025-03-06

**Authors:** Hyeji Lee, Wooheon Kim, Nahyeon Kwon, Chanhee Kim, Sungmin Kim, Joon-Yong An

**Affiliations:** 1 Department of Integrated Biomedical and Life Science, Korea University, Seoul, 02841 Republic of Korea; 2 L-HOPE Program for Community-Based Total Learning Health Systems, Korea University, Seoul, 02841 Republic of Korea; 3 School of Biosystem and Biomedical Science, College of Health Science, Korea University, Seoul, 02841 Republic of Korea; 4 Division of Genome Science, Department of Precision Medicine, National Institute of Health, Cheongju, 28159 Republic of Korea

**Keywords:** Whole-genome sequencing, Biobank, Precision medicine, Multi-omics integration, Population genetics

## Abstract

Large-scale national biobank projects utilizing whole-genome sequencing have emerged as transformative resources for understanding human genetic variation and its relationship to health and disease. These initiatives, which include the UK Biobank, All of Us Research Program, Singapore’s PRECISE, Biobank Japan, and the National Project of Bio-Big Data of Korea, are generating unprecedented volumes of high-resolution genomic data integrated with comprehensive phenotypic, environmental, and clinical information. This review examines the methodologies, contributions, and challenges of major WGS-based national genome projects worldwide. We first discuss the landscape of national biobank initiatives, highlighting their distinct approaches to data collection, participant recruitment, and phenotype characterization. We then introduce recent technological advances that enable efficient processing and analysis of large-scale WGS data, including improvements in variant calling algorithms, innovative methods for creating multi-sample VCFs, optimized data storage formats, and cloud-based computing solutions. The review synthesizes key discoveries from these projects, particularly in identifying expression quantitative trait loci and rare variants associated with complex diseases. Our review introduces the latest findings from the National Project of Bio-Big Data of Korea, which has advanced our understanding of population-specific genetic variation and rare diseases in Korean and East Asian populations. Finally, we discuss future directions and challenges in maximizing the impact of these resources on precision medicine and global health equity. This comprehensive examination demonstrates how large-scale national genome projects are revolutionizing genetic research and healthcare delivery while highlighting the importance of continued investment in diverse, population-specific genomic resources.

## Introduction

The advent of large-scale national genome projects has ushered in a transformative era in genomic research, fundamentally reshaping our understanding of human genetic variation and its relationship to health and disease. These initiatives, characterized by their unprecedented scale and comprehensive approach to data collection, represent a convergence of technological advancement, decreasing sequencing costs, and growing recognition of the value of population-level genetic information. At their core, these projects leverage whole-genome sequencing (WGS) to generate high-resolution genomic data from hundreds of thousands to millions of participants, creating resources that far exceed the scope and detail of previous genetic studies.

The distinctive power of national genome projects lies in integrating comprehensive WGS data with rich phenotypic, environmental, and clinical information [1]. Unlike traditional genetic studies that often focus on specific diseases or traits, these large-scale initiatives enable systematic investigation of the full spectrum of human genetic variation and its impact across multiple health outcomes. This holistic approach has proven valuable for understanding complex diseases where multiple genetic and environmental factors contribute to disease risk and progression. The depth and breadth of WGS data allow researchers to identify rare variants, structural variations, and regulatory elements that might be missed by more targeted approaches such as genotyping arrays or WES. These projects have emerged against growing recognition that existing genomic resources inadequately represent global genetic diversity. Historical biases in genetic research have resulted in datasets predominantly drawn from European populations, limiting the generalizability of findings and potentially exacerbating health disparities. National genome projects from diverse geographic regions, including the UK Biobank [2], All of Us Research Program [3], Singapore’s PRECISE initiative [4], Biobank Japan [5], and the National Project of Bio-Big Data of Korea (NPBBD-Korea) [6], are helping to address this imbalance. By capturing genetic variation across different ancestral backgrounds, these resources enable more inclusive and comprehensive genomic research, ultimately supporting the development of more equitable precision medicine approaches.

However, the scale and complexity of these initiatives present significant challenges. Generating, storing, and analyzing WGS data from large cohorts require substantial computational infrastructure and sophisticated analytical tools. Privacy concerns and ethical considerations surrounding collecting and sharing genetic information necessitate careful governance frameworks. Additionally, integrating genomic data with clinical practice remains a significant challenge, requiring new approaches to data interpretation and clinical decision support. The impact of these projects extends beyond academic research. They catalyze technological innovation in sequencing technologies, bioinformatics tools, and data management systems. Their findings inform drug development, improve disease risk prediction, and advance our understanding of basic biological processes. As these resources mature, they are increasingly used to support clinical applications, from rare disease diagnosis to pharmacogenomic prescribing.

This review examines the methodologies, contributions, and challenges of utilizing WGS data. We provide an overview of key initiatives worldwide, highlighting their distinct approaches and characteristics. We then explore the technological advances that enable these projects, from sequencing technologies to data analysis and storage innovations. The review discusses major scientific discoveries enabled by these resources, particularly in understanding rare variants and disease mechanisms. Finally, we consider the future directions and implications of these projects for advancing precision medicine and global health equity. Through this comprehensive examination, we aim to illuminate how large-scale national genome projects are revolutionizing our approach to genetic research and healthcare while also addressing the challenges and opportunities that lie ahead in maximizing their impact on human health.

## Overview of national genome projects with whole-genome sequencing data

**Fig. 1:**
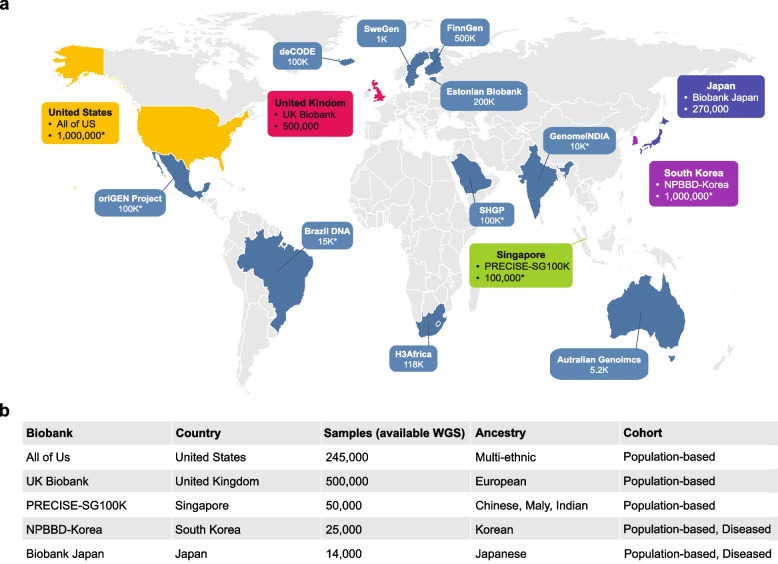
Overview of genomic resources in the national biobanks. **a** Geographical distribution of the biobanks, with the sample numbers representing the total cohort size targeted or achieved by each biobank. Major biobanks possessing large-scale WGS datasets exceeding 10,000 individuals are highlighted. An asterisk (“*”) indicates the targeted cohort size. **b** Detailed information on WGS sample sizes, ancestry composition, and health conditions of the respective biobank datasets were recently disclosed

National biobanks have emerged as critical platforms for advancing genomics research, combining large-scale participant cohorts with WGS across various countries (Fig. 1). By integrating high-resolution WGS data with comprehensive phenotypic, environmental, and clinical datasets, these initiatives enable researchers to uncover the genetic architecture of diseases, identify novel biomarkers, and develop precision medicine strategies tailored to diverse populations. The emphasis on WGS within these national biobanks provides unparalleled insights into genetic variants across different ancestries and establishes a robust foundation for understanding population-specific health trends, improving disease prediction, and fostering equitable healthcare solutions.

The UK Biobank is a large-scale biomedical database that aims to understand the relationship between genetic, environmental, and lifestyle factors in health and disease. It has recruited approximately 500,000 participants aged 40–69 years, with the cohort representative of the general UK population [7]. Among these participants, 452,264 individuals are of European ancestry, accounting for 93.5% of the cohort, while 9229 are of African ancestry, 9674 are of South Asian ancestry, 2869 are of Ashkenazi Jewish ancestry, and 2245 are of East Asian ancestry. The cohort comprises 54% females and 46% males, with a balanced sex ratio enabling robust sex-stratified analyses [8]. The UK Biobank collects extensive phenotypic data through surveys on lifestyle, medical history, and environmental exposures, as well as physical and cognitive assessments and linkage to electronic health records (EHR). This resource includes comprehensive data from healthy individuals and those with various medical conditions. Genomic data generation has been a significant focus of the project, with WGS data available for 490,640 participants, encompassing over 1.1 billion single-nucleotide polymorphisms (SNPs) and approximately 1.1 billion insertions and deletions [9]. The genetic and phenotypic data already available establishes the UK Biobank as one of the most comprehensive resources for population-based health research.

The All of Us Research Program in the United States is designed to drive precision medicine by gathering data from a diverse population to understand better the factors influencing health and disease. As of February 2024, the program has released WGS data for 245,388 participants, with the goal of sequencing over one million individuals [10]. Among participants with WGS data, 77% belong to groups historically underrepresented in biomedical research, including 22% of African or African American ancestry, 18% of Hispanic or Latino ancestry, 2% of Asian ancestry, and 51.1% of European ancestry, along with individuals of mixed or other ancestries. The program ensures gender balance and comprehensive phenotypic data collection, which includes surveys on demographics, lifestyle, family history, and medical history, along with physical measurements such as height, weight, blood pressure, and waist circumference. EHR data are available for over 287,000 participants, and 77% of participants possess both survey data and physical measurements in addition to WGS data [11]. One-fourth of participants have up to 10 years of longitudinal EHR data. Data from wearable devices also enrich the dataset, capturing metrics on physical activity and sleep patterns. Researchers access data via a secure cloud-based platform, which supports detailed analyses. The program’s inclusion of diverse populations addresses long-standing biases in genomic studies and facilitates more inclusive approaches to precision medicine.

Singapore’s National Precision Medicine Programme, PRECISE, aims to transform healthcare by integrating genomic and phenotypic data. The program is divided into three phases, spanning from 2017 to 2027 [4]. In Phase 1, the SG10K_Health cohort was established, consisting of high-quality genome sequencing data from 9770 individuals representative of Singapore’s major ethnic groups: Chinese (58.4%), Indian (21.8%), and Malay (19.5%). Phase 2 expanded to the PRECISE-SG100K cohort, a longitudinal study involving over 100,000 individuals, integrating existing cohorts and newly recruited participants to reflect the country’s ethnic diversity better. Data collection includes comprehensive phenotypic information such as cardiovascular and metabolic health markers, advanced imaging tests, nutrition, and dietary habits. WGS is being conducted for all participants, and additional genomic data, including WES and SNP [3] arrays, are being generated for selected samples. Multi-omics efforts encompass transcriptomics, proteomics, metabolomics, epigenomics, microbiome analyses, and advanced imaging, providing a holistic view of biological processes. Phase 3, planned for 2024–2027, aims to scale the cohort to 500,000 participants, enhancing statistical power for studying genetic and environmental factors influencing health.

BioBank Japan (BBJ) was established to support genetic research on 51 common diseases affecting the Japanese population [12, 13]. Approximately, 200,000 participants were enrolled in the first phase (2003–2008), followed by 70,000 participants in the second phase (2012–2017). The cohort’s gender distribution is relatively balanced, with 53.1% male and 46.9% female participants. BBJ has collected detailed phenotypic data, including general clinical information such as smoking and drinking habits, anthropometric measurements, personal and family medical histories, and disease-specific data. WGS data are available for 14,000 individuals, and SNP array genotyping has been performed on 270,000 participants across two cohorts [13]. BBJ has made significant advancements in multi-omics research, completing metabolomic analyses on 4000 individuals and planning additional analyses for 60,000 participants. Proteomic data have been generated for 3000 individuals, with another 3000 samples undergoing analysis. By integrating genomic, phenotypic, and multi-omics data, BBJ provides valuable insights into disease mechanisms and precision medicine applications.

In South Korea, the NPBBD-Korea is a national R&D initiative to establish an integrated bio-big data resource for 1 million Koreans over 9 years, from 2024 to 2032. Under this project, personal data will be gathered—upon consent—from participants, including biospecimens, clinical information, medical records, public institution data, personal health data, and genomic and other omics data. During Phase 1 (2024–2028), 772,000 individuals will be recruited (47,000 with rare diseases, 140,000 with severe/cancer diseases, and 585,000 from the general population) to collect clinical and public data. Among them, 240,000 people—including 47,000 with rare diseases, 140,000 with severe/cancer diseases, 38,000 general individuals with chronic conditions, and 15,000 general control subjects—will have blood samples taken for 30 × WGS. Cancer patients will additionally provide blood, urine, and tissue samples, enabling the production of 60 × WGS data for 41,000 samples across 13 cancer types and multi-omics data (transcriptome, proteome, metabolome) for 3000 samples in five cancer types. During Phase 2 (2029–2032), clinical and public data from 228,000 individuals (23,000 with rare diseases, 80,000 with severe/cancer diseases, and 125,000 from the general population) will be collected. WGS will be analyzed for 103,000 people with rare/severe/cancer diseases, 160,000 general individuals with chronic diseases, 40,000 general individuals with severe diseases, and 30,000 general control subjects. Across both phases, the project aims to finalize clinical and public data collection for 1 million participants and generate WGS data for 550,000 individuals, ultimately establishing a comprehensive biobank. The resulting data will be available to researchers starting in 2026. The pilot project of the NPBBD-Korea encompassed several genomic cohorts, including rare diseases, autism spectrum disorder (ASD), and lung cancer, as well as large-scale general population cohorts from the Korean Genome and Epidemiology Study (KoGES) [14] and Ulsan citizens [6, 15]. WGS data collection began recently under the pilot project of the NPBBD-Korea, with sequencing completed for 25,000 individuals, including ~ 15,000 rare disease cases, ~ 3000 other disease cases, and ~ 7000 healthy individuals [16].

In addition, several other initiatives are advancing our understanding of genetic diversity and its impact on health. The Estonian Biobank has recruited 200,000 participants, representing the demographic structure of Estonia, with 83% being ethnic Estonians [17]. The project has collected extensive phenotypic and genomic data, including 2244 high-quality WGS and multi-omics datasets such as transcriptomics, metabolomics, and epigenomics [18, 19]. The GenomeAsia 100 K Project focuses on the genetic diversity of Asian populations, generating high-quality WGS data for 1267 samples across diverse ethnic groups from India, Malaysia, Korea, and beyond [20]. India’s GenomeIndia project has sequenced 2515 samples to understand disease risks, rare disorders, and pharmacogenomics within the Indian population [21]. Japan’s Tohoku Medical Megabank Organization has sequenced 8380 high-quality WGS samples and genotyped for 150,000 individuals, integrating multi-omics data to study gene-environment interactions [22]. The Swedish SweGen project has constructed a comprehensive map of genetic variation within Sweden, with whole-genome sequencing data of 1000 Swedish individuals [23]. While varying in scale and focus, these projects collectively enhance the global effort to understand genetic diversity and advance precision medicine.

## Technological advances in national genome projects

WGS data from national biobank projects are extremely large, encompassing vast genetic information from thousands or even millions of individuals. Managing and analyzing such massive datasets present significant challenges that require advanced technological solutions. To address these challenges, various technological advancements and pipelines have been made to process and analyze big genomic data efficiently (Table 1). These developments include robust variant calling tools, innovative methods for creating multi-sample VCFs, optimized data representation and storage formats, cloud-based computing environments, and advanced downstream analysis tools and methodologies.

**Table 1:** Pipelines and bioinformatics tools utilized in genomic resources in the national biobank

	**UK Biobank**	**NPBBD-Korea**	**PRECISE**	**BBJ**	**All of Us**
**Variant calling**	GATK DRAGEN (FPGA-accelerated)	GATK	GATK	GATK	DRAGEN GATK DeepVariant (deep learning-based precision)
**Multi-sample VCF**	GATK (GenotypeGVCFs) DRAGEN (DRAGEN Iterative gVCF Genotyper for scalability) Graphtyper	GATK (GenotypeGVCFs)	GATK (GenotypeGVCFs)	GATK (GenotypeGVCFs) Graphtyper	Genomic Variant Store (GATK based) Glnexus
**Data representation & storage**	BAM/CRAM Sparse VCF	BAM gVCF	BAM/CRAM Sparse VCF	BAM/CRAM Dense VCF	BAM/CRAM Sparse VCF (Hail matrix, VDS)
**Computing environment**	Cloud-based RAP with DNAnexus and AWS	KISTI National Supercomputing Center (https://www.ksc.re.kr/eng/index/main)	RAPTOR (Research Assets Provisioning and Tracking Online Repository)	Local HPC for server-based analysis	Cloud-based workbench (Google Cloud Platform for large-scale analysis)
**Data management system**	“Category-field”-based data structure	DRC and RDR-CDR system	-	-	GIMS
**Data access system**	Tier system, paid for all tiers	Tier system, free for all tiers	Tier system, free for all tiers	Tier system, free for all tiers	Tier system, free for all tiers

### Tools for variant calling

The Genome Analysis Toolkit (GATK) pipeline has long been the standard for variant calling, integrating BWA-MEM for aligning sequencing reads to a reference genome and the GATK for variant calling [24, 25]. This workflow is extensively utilized by prominent projects such as the UK Biobank [9], NPBBD-Korea [26], PRECISE [4], and BBJ [27] due to its reliability and precision. However, the computational intensity and slower processing times associated with GATK present significant challenges when scaling to ultra-large datasets, limiting its efficiency for expansive genomic studies.

To overcome these limitations, alternative variant calling tools have gained significant traction. DRAGEN employs FPGA-based hardware acceleration to enhance processing speeds and reduce latency substantially [28]. This hardware-accelerated approach enables DRAGEN to manage large-scale datasets more efficiently, making it an ideal choice for extensive projects such as the All of Us Research Program [10] and UK Biobank [9]. DRAGEN accelerates variant calling and integrates other genomic analysis steps, including alignment, duplicate marking, and base quality score recalibration, offering a comprehensive and streamlined workflow optimized for both speed and accuracy. In addition to DRAGEN, Sentieon and DeepVariant represent significant advancements in variant calling methodologies [29, 30]. Sentieon accelerates GATK workflows by providing fully compatible algorithms that improve speed and scalability without compromising accuracy, making it valuable for projects aiming to scale their variant calling processes [31]. DeepVariant utilizes deep learning techniques to enhance variant detection precision by distinguishing true variants from sequencing errors [30]. These advancements optimize variant calling processes, ensuring large-scale genomic data can be analyzed accurately and efficiently.

### Methods for creating multi-sample VCF

Managing multi-sample VCF files is crucial for large-scale genomic studies, where integrating data from numerous samples is essential. Creating multi-sample VCFs facilitates cross-sample comparison of genetic variants, aids in the identification of population-level allele frequencies and rare variants, and increases the confidence of variant calls through aggregated evidence from multiple samples [32]. Two primary approaches to generating multi-sample VCFs are joint calling [33] and aggregation. Each approach employs distinct methodologies and presents unique trade-offs, making it suitable for different project needs.

Joint-calling involves analyzing multiple samples simultaneously to call variants. This method enhances accuracy by leveraging the shared genetic information among samples, allowing for more precise detection of rare variants. By considering variants across the cohort, joint calling reduces false positives and ensures consistency in variant calls. The GATK GenotypeGVCFs is a widely used tool for this purpose. Employed in projects like the NPBBD-Korea, PRECISE, and Biobank Japan, GATK GenotypeGVCFs enable efficient genotyping of multiple samples together, improving the reliability of variant detection in large datasets. Graphtyper is another joint-calling tool that uses a graph-based approach to model genetic variation more effectively than traditional linear methods [34]. Representing complex genetic structures within a variation graph enhances variant calling accuracy, especially in regions with high diversity or structural variation. Used in the UK Biobank alongside GATK, Graphtyper improves the detection of variants that linear approaches might miss.

Aggregation involves integrating variants called independently in individual samples. This approach offers ease and flexibility in parallel processing, as each sample can be processed separately without simultaneous analysis. It is particularly advantageous for ultra-large cohorts where joint-calling becomes computationally prohibitive due to extensive time and resource requirements. By shifting to aggregation, researchers can efficiently manage and analyze large datasets. A significant benefit is its ability to solve the N + 1 problem—the challenge of adding new samples to an existing dataset without reprocessing the entire cohort. Aggregation allows seamless incorporation of new samples by merging their individually called variants with existing data, thus avoiding the need for complete reanalysis. Tools like the DRAGEN Iterative gVCF Genotyper (IGG) used in the UK Biobank, the Genomic Variant Store (GVS) developed by the All of Us Research Program, and GLnexus exemplify this approach. DRAGEN IGG enables efficient processing of large genomic datasets by iteratively processing gVCF files from individual samples and aggregating the results, significantly reducing computational time while maintaining high accuracy in variant detection. GVS provides a scalable solution for managing, storing, and accessing aggregated variant data from vast samples without the computational demands of joint-calling methods. Similarly, GLnexus can be used in this aggregation method, efficiently merging gVCF files from individual samples into a joint genotyped multi-sample VCF.

In the context of genomic research, the choice between joint-calling and aggregation methods for creating multi-sample VCFs depends on the specific needs and constraints of the project. Joint calling is preferred when the highest possible accuracy is required and computational resources are sufficient to handle the simultaneous analysis of multiple samples. It is particularly beneficial for detecting rare variants and ensuring consistency across the dataset. Aggregation offers a practical alternative for projects involving ultra-large cohorts or when new samples are continually added to the dataset. It provides scalability and flexibility, allowing researchers to efficiently manage and analyze extensive genomic data without the prohibitive computational costs associated with joint calling.

### Changes in data representation and storage

Managing vast WGS data volumes has driven significant innovations in data representation and storage methodologies. Due to their superior compression capabilities, CRAM (Compressed Reference-oriented Alignment Map) files are increasingly replacing traditional BAM (Binary Alignment/Map) files [35]. By compressing alignment data relative to a reference genome, CRAM achieves about a 50% reduction in storage requirements compared to BAM files. This significant efficiency is particularly beneficial for large-scale projects where storage costs and data transfer speeds are critical. CRAM optimizes storage by eliminating redundancy in the alignment data without compromising data accessibility.

For variant data, VCF remains the standard for storing variant calls [36]. However, due to their substantial size, traditional dense VCFs become unwieldy with large cohorts [37]. To address this, sparse VCF formats have been developed, focusing solely on essential variant information to reduce data size and enhance processing efficiency. By adopting sparse VCFs, researchers can efficiently manage and analyze large-scale genomic data, significantly improving storage efficiency and processing performance for more effective and scalable genomic analyses. Sparse VCF implementations, such as Hail Variant Dataset (VDS), utilized by the All of Us program, and DRAGEN IGG multi-sample VCF, employed by UK Biobank, facilitate efficient storage and rapid access to variant data [10]. Hail VDS leverages the Hail framework to provide a scalable and efficient representation of variant data, enabling rapid querying, filtering, and analysis across large cohorts by optimizing data storage and access patterns [37]. DRAGEN utilizes a compact representation of multi-sample variant calls, storing genotype information in a highly efficient format that facilitates fast access and analysis without the overhead associated with traditional VCF formats. Tools like Savvy and Sparse Project VCF also optimize data management by converting dense VCF files into sparse formats, retaining essential variant information while reducing redundancy [37–39]. This makes large-scale genomic datasets more manageable without compromising data integrity.

### Computing environments

The transition from local server-based data analysis to cloud-based environments has been pivotal for managing large-scale genomic projects [40]. Cloud platforms offer scalable computational resources, integrated storage solutions, and specialized analytical tools tailored for genomics. This addresses the challenges of processing and storing vast amounts of data from ultra-large cohorts, enabling efficient execution of computationally intensive tasks like whole-genome sequencing without significant hardware investments. By leveraging cloud infrastructure, researchers can focus on scientific inquiry rather than logistical hurdles, making cloud computing essential for handling the demands of modern genomic research. Notably, projects such as NPBBD-Korea and BBJ primarily utilize local server-based approaches. At the same time, UKBB, All of Us, and PRECISE rely on cloud-based environments to manage and analyze genomic data.

The UK Biobank Research Analysis Platform (RAP) exemplifies this shift by providing a cloud-based environment specifically designed for UK Biobank data. Built on DNAnexus and Amazon Web Services (AWS) infrastructure, RAP allows researchers to perform complex analyses directly within the cloud. This eliminates the need for extensive local computational infrastructure and facilitates seamless collaboration across institutions, enabling researchers to access and analyze data efficiently from any location. Similarly, the All of Us Workbench operates on the Google Cloud Platform (GCP), offering robust access to vast datasets and integrating various analytical tools to support comprehensive genomic and phenotypic studies [10]. The workbench leverages GCP’s scalable infrastructure to support large-scale data processing tasks, including real-time data querying, machine learning applications, and interactive data exploration. Additionally, it ensures data security and privacy by implementing stringent access controls and encryption protocols, thereby safeguarding sensitive genomic information while maintaining accessibility for authorized researchers.

Cloud providers like AWS, GCP, and Microsoft Azure offer specialized genomics services that enhance large-scale genomic data analysis. These services—AWS Genomics Workflows, DeepVariant, and Microsoft Azure Genomics—provide scalable tools for variant calling, alignment, and computational customization [30]. Platforms like Terra facilitate GATK workflows for collaborative research and efficient whole-genome sequencing analysis. Additionally, containerization technologies (e.g., Docker, Singularity) and workflow management systems (e.g., Nextflow, WDL) automate and streamline genomic pipelines, improving efficiency and scalability across different computational environments.

### Downstream analysis tools and methods for biobank-scale genomic resources

Advanced analysis tools like Hail and Glow have significantly enhanced the processing and interpretation of large-scale genomic data by leveraging distributed computing frameworks such as Apache Spark. Hail (https://hail.is/) enables complex analyses—including association studies, population genetics, and variant annotation—on datasets with millions of variants and tens of thousands of samples without performance bottlenecks, offering a user-friendly API compatible with Python and Scala for developing custom analysis pipelines. Glow (https://projectglow.io/) builds upon Apache Spark with optimized data structures and algorithms specifically designed for genomic data, enhancing tasks like variant filtering, annotation, and quality control. By abstracting the complexities of distributed computing and integrating with cloud-based environments, Glow allows researchers to focus on their analyses without managing the underlying infrastructure, making it valuable for national genome projects. Both tools reduce computational overhead, enable faster processing of large-scale genomic data, and support more sophisticated analyses.

Innovative methods like region-based association testing and pre-subsetting of genomic regions have been developed specifically for big data analysis to analyze large genomic datasets efficiently. These techniques enable more targeted and parallel processing of BAM and VCF files by dividing the genome into predefined sections and conducting independent association tests within each region. This design significantly reduces computation time and enhances the precision of identifying genetic variants associated with traits or diseases by focusing computational resources on relevant areas. By optimizing large-scale data analysis, these approaches improve the resolution and accuracy of genetic association studies, facilitating rapid insights into the genetic basis of various conditions.

### Data management and access

Managing large-scale biobank data requires a robust and systematic approach, as effective data quality management and version control are essential for ensuring reliability and usability. To achieve this, each biobank systematically organizes and manages metadata generated during data production and analysis, making it easily accessible to users. For example, the UK Biobank employs a “category-field”-based data structure system for organized metadata verification, such as data quality indicators and versioning [41]. Similarly, the All of Us has established a Data and Research Center (DRC) to oversee data management and access control. Within this framework, raw data is stored in the Raw Data Repository (RDR), while processed and refined data is housed in the Curated Data Repository (CDR), creating a two-tiered storage system for greater efficiency [42]. NPBBD-Korea utilizes the Genomic Information Management System (GIMS) developed by the Korean Bioinformation Center [16]. This system ensures systematic management of all metadata and quality control indicators throughout the entire process, from data production to analysis, further enhancing data reliability and usability.

Given the complexity and sensitivity of biobank data, access to these datasets is typically governed by tiered access systems designed to balance usability, security, and privacy. The UK Biobank provides data access through a tiered system that balances usability and security. Tier 3, the highest level, includes comprehensive datasets such as genomic sequences and imaging data, accessed via secure platforms like RAP for data viewing and “ukbfetch” for downloads [43]. Researchers must meet strict requirements, including project approval, institutional agreements, and compliance with the material transfer agreement. The All of Us Research Program offers secure, cloud-based data access through a tiered system designed to protect privacy [44]. Public access data is freely available with minimal reidentification risk. Registered access data, with explicit identifiers removed, requires registration and compliance with the Data User Code of Conduct. Controlled access data includes sensitive phenotypic and genomic information, requiring additional review and approval. SG10K_Health facilitates data access through the RAPTOR platform under a structured governance system [45]. Researchers must submit detailed requests to the National Precision Medicine Data Access Committee and conduct analyses within secure workspaces. Raw data remains protected, and only approved summarized results may be exported.

BBJ provides access through its internal and public databases, including the NBDC Human Database and the AMED Genome Group Sharing Database [13]. Its three-tiered system includes controlled-access data, which requires approval; group-shared data, available for academic studies; and unrestricted-access data, which is openly available but strictly limited to research use. NPBBD-Korea organizes data within the Korean BioData Station into four tiers based on sensitivity [46]. Tier 1 includes general information freely accessible online. Tier 2 offers non-identifiable data with monitored downloads. Tier 3 requires IRB and committee approval for data with moderate re-identification risks. Tier 4 includes highly sensitive clinical or genetic data requiring additional review and approval.

### Security issues

Biobanks face significant challenges in ensuring data security, protecting participant privacy, and complying with ethical and legal standards. A primary concern is data reidentification, where anonymized datasets can be matched with external information to reveal identities [47]. Advanced anonymization techniques, dynamic data processing, strict access controls, and real-time monitoring are critical to mitigating this risk. Cybersecurity is another major issue, as biobanks are vulnerable to attacks that could compromise sensitive data. Measures such as encryption, multifactor authentication, and controlled virtual environments, like those implemented by the UK Biobank, enhance data security. Regular cybersecurity drills and proactive strategies help address emerging threats.

Ethical and legal compliance is essential for maintaining public trust and adhering to research standards [48]. Programs like All of Us use “data passports” to facilitate international research while adhering to local protocols. Regular audits and transparent policies are essential to fostering public trust. Informed consent remains vital, requiring clear communication about data usage and participant rights supported by electronic consent systems.

While data sharing is critical for advancing research, it carries risks of misuse [49]. Tiered access systems, strict data use agreements, and secure platforms like SG10K_Health’s RAPTOR system balance accessibility with confidentiality. Addressing these challenges requires technological innovation, strong ethical frameworks, and active participant engagement to protect biobank data and support impactful research.

## What have we discovered and learned from the genomic studies of the biobank projects?

**Fig. 2:**
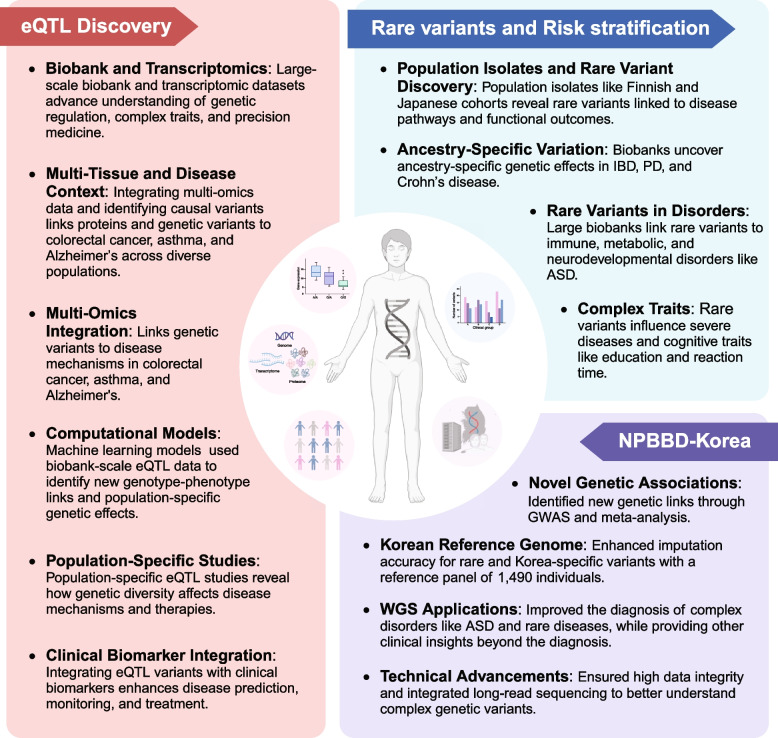
Key discoveries and insights from biobank-based genomic studies. eQTLs have revolutionized our understanding of how genetic variation influences gene expression, providing a crucial molecular bridge between genomic variants and complex phenotypes. Biobank datasets have transformed genetic research by enabling large-scale rare variant discovery and more nuanced approaches to disease risk stratification by integrating rich phenotypic, environmental, and biomarker data. Additionally, the NPBBD-Korea has provided various biological and clinical insights through high-quality WGS data and aims to improve health outcomes in East-Asian populations through continuous data expansion

Large-scale national biobank projects have yielded numerous groundbreaking discoveries that advance our understanding of human genetics and disease mechanisms (Fig. 2). These findings span multiple areas, from identifying expression quantitative trait loci (eQTLs) that illuminate gene regulation to discovering rare variants that contribute to disease risk. In addition, the breadth of phenotypic information and population-specific cohorts has enabled the delineation of genetic diversity and rare variants conferring risk to diseases.

### Discovery of eQTL loci using biobank genomic resources

eQTLs have revolutionized our understanding of how genetic variation influences gene expression, providing a crucial molecular bridge between genomic variants and complex phenotypes. The emergence of mega-scale biobank projects and extensive transcriptomic datasets enables systematic mapping of eQTLs across diverse populations, tissues, and disease states, facilitating the discovery of novel regulatory mechanisms, deepening our understanding of disease biology, and advancing precision medicine strategies. Early studies leveraging the UK Biobank and other large resources provided foundational insights into the genetic regulation of gene expression and complex traits, establishing the groundwork for more sophisticated analyses. For instance, Barbu et al. utilized the UK Biobank data to develop eQTL scores associated with psychiatric disorders, revealing significant connections between these scores and in vivo brain structural connectivity [50]. At the same time, Ward et al. conducted a comprehensive genome-wide association study (GWAS) of mood instability using the UK Biobank, identifying 46 distinct loci linked to nervous system pathways and expanding our understanding of psychiatric phenotypes [51].

The integration of multi-tissue and disease context analyses has substantially advanced our understanding of regulatory networks and disease mechanisms. Gamazon et al. synthesized eQTL data from 44 tissues, including biobank-derived samples, to explore tissue-specific and shared regulatory networks underlying various traits, providing crucial insights into the tissue-specific nature of gene regulation [52]. This work was complemented by Tachmazidou et al., who combined eQTL analyses with therapeutic target identification in osteoarthritis, uncovering disease-specific pathways that informed precision medicine approaches and demonstrated the practical applications of eQTL research in drug development [53]. Further expanding these insights, a transcriptome-wide association study identified genetic loci associated with calcific aortic valve stenosis, demonstrating the power of large-scale genotype-transcriptome data integration in elucidating disease mechanisms and highlighting the potential for identifying novel therapeutic targets through integrated analyses [54].

Recent advances in multi-omics integration and causal variant identification have significantly enhanced our understanding of complex diseases across diverse populations. A comprehensive study of colorectal cancer risk combined plasma proteome data with genome-wide summary statistics from FinnGen, UK Biobank, and multiple GWAS datasets, successfully identifying 13 proteins and shared causal variants linked to colorectal cancer development and progression [55]. This multilayered approach to disease investigation was further exemplified in asthma research, where investigators utilized eQTL data from peripheral blood mononuclear cells and nasal samples to identify regulatory variants that modulate systemic and airway-specific gene expression, providing insights into tissue-specific disease mechanisms [56]. Similarly, in Alzheimer’s disease research, the integration of eQTL data with cerebrospinal fluid biomarker profiles highlighted crucial loci, including APOE and TMEM106B, thereby refining our understanding of AD pathogenesis and identifying potential therapeutic targets through the combination of genetic and molecular approaches [57].

The development of innovative computational models has substantially expanded the utility of biobank-scale eQTL resources, enabling more sophisticated analyses of complex genetic relationships. The DeepGAMI model successfully integrated eQTLs and gene regulatory networks from PsychENCODE, ROSMAP, and GTEx, revealing novel genotype–phenotype relationships in brain diseases such as schizophrenia and AD while demonstrating the power of machine learning approaches in understanding complex neurological conditions [58]. These computational advances have been particularly valuable in analyzing UK Biobank data, where researchers have uncovered pleiotropic variants affecting both blood traits and cancer risk, with immune-related pathways emerging as central regulatory hubs in disease development [59]. These sophisticated analytical approaches have also facilitated the identification of population-specific genetic effects, as demonstrated by studies in the SIREN cohort for stroke in African populations, which have revealed unique variants with protective or pathogenic effects on disease outcomes [60].

Population-specific studies have emerged as a crucial frontier in eQTL research, highlighting the importance of genetic diversity in understanding disease mechanisms and developing targeted therapeutic approaches. The Qatar Biobank has made significant contributions by linking eQTL loci to Mendelian disorders, emphasizing the significance of population-specific allele frequencies in rare variant interpretation, and demonstrating how genetic architecture can vary across different populations [61]. Similarly, integrative analyses in lung cancer, using nasal and bronchial samples from the CRUKPAP cohort, have identified germline variants affecting tissue-specific gene expression, immune pathways, and the influence of smoking exposure, showcasing the importance of considering both genetic and environmental factors in disease development [62]. Work in East Asian populations has further expanded our understanding of population-specific effects, particularly in colorectal cancer tissues, where researchers employed eQTL mapping and chromatin interaction data to uncover novel regulatory variants influencing PANK1 expression and other cancer-related pathways [63].

The integration of eQTL variants with clinical biomarkers represents a significant advance in translating genetic discoveries into clinical applications, with implications for disease prediction, monitoring, and treatment optimization. Multi-omics studies combining proteome-wide and transcriptome-wide data from CKDGen, UK Biobank, and FinnGen have identified key proteins associated with chronic kidney disease progression, providing new insights into disease mechanisms and potential therapeutic targets [64]. These findings have been complemented by investigations of immunoglobulin glycosylation traits using TwinsUK and QMDiab datasets, which have revealed conserved genetic architectures underlying immune regulation and demonstrated the importance of considering posttranslational modifications in genetic studies [65]. The analysis of plasma metabolites in the GCAT and Genomes for Life cohort and large European datasets has further expanded our understanding of how eQTL loci connect with cardiovascular risk factors, particularly highlighting the roles of genes such as PCSK9 and CELSR2 in lipid metabolism and cardiovascular disease development [66]. The examination of indigenous populations, exemplified by studies of the Tiwi community in Australia, has demonstrated how population-specific biobanks can uncover unique variants influencing chronic kidney disease that may be absent or rare in other populations, underscoring the crucial importance of expanding eQTL discovery efforts beyond traditional cohorts to capture the full spectrum of human genetic diversity and its impact on disease susceptibility and progression [67]. These diverse approaches to biomarker integration and population-specific analysis highlight the growing sophistication of eQTL research and its increasing relevance to clinical practice and precision medicine initiatives.

### Leveraging biobank data for rare variant discovery and risk stratification

Biobank datasets have revolutionized genetic research, enabling large-scale rare variant discovery and more nuanced approaches to disease risk stratification. These extensive resources, often coupled with rich phenotypic, environmental, and biomarker data, provide unprecedented opportunities to deepen our understanding of complex traits and diseases. By integrating insights from population isolates, diverse ancestral groups, and well-defined clinical cohorts, researchers can reveal previously inaccessible genetic variants that inform more precise medical interventions.

One of the key advantages of biobank studies lies in exploring population isolates, where historical bottlenecks and limited gene flow have shaped distinct genetic architectures. These demographic factors concentrate on low-frequency and rare variants that often remain undetectable in more heterogeneous cohorts. Kurki et al. utilized the Finnish FinnGen resource and identified deleterious alleles enriched in the Finnish population [17]. This underscores the value of such resources for uncovering disease mechanisms. Similarly, Nagasaki et al. used deep whole-genome sequencing in Japanese cohorts to identify rare variants enriched in disease-relevant pathways [68]. These findings underscore the benefits of incorporating diverse population backgrounds into large-scale genomic analyses. Biobanks have also facilitated unprecedented examinations of protein-coding variants and allowed researchers to link these genomic changes directly to functional consequences. Sun et al. identified clinically relevant disease-specific loci from the UK Biobank [69], while the latest study linked genetic variation to blood and urine biomarkers that inform disease prediction and prevention [70]. Large-scale datasets further enable structural variant discovery, such as copy-number variants (CNVs). Using data from the National Bio-Big Data Project, the UK Biobank, and the Estonian Biobank [71], researchers uncovered 73 disease-associated CNVs, connecting specific genomic regions to conditions including epilepsy, hypertension, and chronic kidney disease.

A critical strength of biobank-driven research is its capacity to address ancestry-specific variation and the complexity of comorbid conditions. In a study investigating IBD and PD comorbidity using WGS data [72], high-impact rare variants in genes like LRRK2 and IL10RA were implicated in overlapping disease pathways [73]. Another investigation of Crohn’s disease risk alleles in African American cohorts demonstrated that variants common in Europeans were less frequent in African Americans [74], emphasizing that genetic effects differ across populations. Similarly, rare variant association studies have identified population-specific effects in diverse samples, reinforcing the importance of inclusive and cross-ancestry research strategies.

As biobanks scale to hundreds of thousands of participants, they afford systematic searches for rare variants associated with both common and less prevalent disorders. Exome and genome sequencing of large cohorts has linked rare variants to conditions such as beta-thalassemia, congenital factor XI deficiency, and immune thrombocytopenic purpura [75]. Additional studies identified rare coding variants influencing complex traits like hyperlipidemia [76] and demonstrated that rare and common variants often converge on the same biological pathways [77–79]. The exome study of Australian autism families showed the oligogenic inheritance of de novo and rare inherited variations associated with autism and showed the enrichment of risk variant genes in the synaptic process [80], consistent with the major autism neurobiology [81, 82]. Such convergence enhances our understanding of disease biology and helps refine therapeutic strategies. Beyond traditional disease endpoints, rare variants also shape complex traits, including cognitive function. Analyzing WES data from nearly half a million UK Biobank participants [83], researchers identified protein-truncating and damaging missense variants that significantly affect educational attainment and reaction time. These findings demonstrate that rare variant discovery can illuminate the genetic architecture of a broad spectrum of phenotypes, from severe diseases to subtle cognitive measures.

### Latest findings with the National Bio-Big Data of Korea

NPBBD-Korea has been utilized to identify novel genetic associations in the Korean population. The recent study conducted a GWAS on 76 phenotypes using data from the KoGES and uncovered 122 novel associations with these phenotypes [84]. A meta-analysis combining 32 phenotypes from KoGES and BBJ yielded 379 additional novel associations and enhanced the predictive power of polygenic risk scores. Publicly available summary statistics for the 76 KoGES GWAS phenotypes contribute to a deeper understanding of the genetic landscape in East Asian populations. This work underscores the importance of population-specific databases, which enhance genetic research, imputation accuracy, and the discovery of rare variants [85]. The establishment of the Korean Reference Genome, a part of the NPBBD-Korea pilot dataset, has substantially improved imputation accuracy, especially for variants that are rare or unique to the Korean population [15]. A study utilizing data from 1490 individuals demonstrated that a Korean-specific reference panel outperforms existing panels, thereby strengthening the foundation for future population genetics, disease association studies, and precision medicine approaches [85]. In addition to population-level insights, the NPBBD-Korea has led to significant progress in understanding the genetic underpinnings of complex disorders, such as ASD and neurodevelopmental disorders. WGS data from the project revealed de novo mutations that disrupt chromatin interactions in ASD, contributing to altered gene expression and lower IQ in affected individuals [86], as well as short-tandem repeat expansion associated with low adaptability in ASD [87]. These studies expand additional genetic factors of ASD risk beyond de novo and rare coding variants [88, 89]. Similarly, the application of trio-based WGS analysis in children with neurodevelopmental disorders achieved a diagnostic yield of 33%, demonstrating the power of WGS in uncovering structural, intronic, and other noncoding variants that elude conventional exome sequencing approaches [90].

The value of WGS extends further into rare disease diagnosis, as evidenced by studies focused on Charcot–Marie–Tooth disease [91], inherited retinal diseases [92, 93], and Marfan syndrome [94]. These investigations have repeatedly shown that WGS can detect complex variant types such as intronic, structural, and Alu insertions that are not readily identified by exome sequencing or targeted methods. By revealing these elusive genetic factors, researchers are improving the diagnostic yield and refining clinical management strategies for rare, heterogeneous, and previously unsolved cases. Beyond rare diseases, NPBBD-Korea research also informed other clinically relevant areas. WGS analysis of the Korean population has provided critical data on blood group genotypes, enabling more accurate prediction of transfusion-related phenotypes, including the prevalence of rare blood types [95]. In cardiomyopathy, WGS-based variant classification and pathogenicity assessments of MYH7 variants have supported precision diagnostics and potential therapeutic interventions [96]. Additionally, sex differences in genetic burden for ASD have been explored using the largest East Asian autism WGS dataset, revealing a higher de novo protein-truncating variant burden in females and offering new perspectives on sex-differential liability and phenotype severity [26].

The impact of data integrity and advanced sequencing technologies has also been a critical focus. Studies have emphasized the importance of DNA quality in ensuring reliable genomic data, correlating higher genomic quality numbers with better sequencing depth and accuracy [97]. Moreover, the integration of long-read sequencing approaches has been crucial for understanding complex variants, epigenetic modifications, and the intricate genomic architecture underlying conditions like neuronal intranuclear inclusion disease [98].

Collectively, the work under the NPBBD-Korea umbrella highlights the transformative potential of bio-big data when integrated with WGS, artificial intelligence, and collaborative international efforts [99]. Large-scale data collection, including clinical and multi-omics datasets, will provide an invaluable resource for refining AI and machine learning models, enabling precise disease prediction, novel biomarker discovery, and targeted therapeutic strategies. This data expansion will also accelerate cancer and rare disease research by facilitating the identification of genetic causes, biomarker development, and innovative treatment approaches. Furthermore, integrating this data with global genomic databases will drive international collaboration, advancing our understanding of complex diseases and expediting the development of safer, more effective drugs tailored to diverse populations, including Korean-specific genetic profiles. By fostering an environment where large-scale, population-specific genomic resources are readily available, NPBBD-Korea is poised to significantly influence global genomics, enhance precision medicine, and improve health outcomes across diverse populations.

## Discussion

National genome projects leveraging WGS have emerged as powerful engines driving modern genetic research and precision medicine. By moving beyond the limitations of genotyping arrays and WES, WGS enables a comprehensive characterization of genomic diversity, encompassing both coding and noncoding regions, structural variants, and ultra-rare population-specific alleles. This all-encompassing approach enhances our fundamental understanding of human genetic variation and paves the way for more effective disease prevention, diagnosis, and therapy.

One of the most transformative aspects of WGS-based national projects is their capacity to illuminate the full spectrum of rare variants. Historically elusive, these variants often profoundly affect disease risk and phenotypic diversity, yet they remain underrepresented in traditional studies. The enhanced resolution of WGS has brought to light previously hidden population-specific variants, as demonstrated by the Tohoku Medical Megabank Project [68], and rare noncoding variants implicated in human traits such as height [100]. These findings highlight the importance of moving beyond coding regions, as intronic and intergenic variants can influence gene regulation and disease pathology. Such discoveries challenge long-held assumptions that common variants explain most heritable risk and underscore [15] the necessity of exploring the entire genomic landscape. Addressing the historical bias toward European ancestry populations is another critical outcome of inclusive national genome projects. By capturing genomic data from diverse populations, these initiatives advance our understanding of the genetic architecture of complex traits across ancestries. Underrepresented groups stand to benefit substantially, as inclusive genomic datasets can improve the accuracy of polygenic risk scores, uncover ancestry-specific disease variants, and inform tailored medical interventions. In a globalized world, this shift toward inclusivity is a scientific and ethical imperative, ensuring that genomic medicine will be equitable and globally relevant.

WGS-powered national biobanks also serve as a springboard for new frontiers in precision medicine. Detailed genetic data can be integrated with clinical, environmental, and lifestyle information, driving the development of individualized risk assessments and targeted therapies. Polygenic risk scores derived from WGS data have already shown promise in predicting conditions like coronary artery disease and type 2 diabetes, as well as severe outcomes in global health crises such as COVID-19 [101]. As new genomic technologies and analytical methods emerge, these predictive models will become increasingly accurate, guiding clinicians in implementing prevention strategies and personalized treatment plans [102]. The exploration of noncoding regions represents another major advance enabled by WGS [103]. While WES provides valuable insights, it covers only a small fraction of the genome, overlooking regulatory elements that can drive disease through subtle modulation of gene expression. Studies like OxClinWGS, which identified structural and deep intronic variants contributing to diagnostic yield [104], reinforce the value of a truly genome-wide perspective. Such comprehensive analyses can inform therapeutic target discovery, revealing how regulatory networks and epigenetic factors influence disease processes. Furthermore, a proteomic dataset will be useful for integrating the WGS data and prioritizing phospho-kinase targets in cancers [105, 106] or complex disorders [107]. Moreover, proteogenomic approaches will yield a more holistic understanding of gene function, enabling the identification of protein quantitative trait loci or proteogenomic biomarkers that can inform drug development and preventive strategies.

Despite these advances, challenges remain. The sheer scale of national genome projects presents computational, logistical, and ethical hurdles. Robust bioinformatic infrastructure and standardized methods are needed to manage, store, and analyze the deluge of data. Ethical considerations around data sharing, privacy, and consent must be carefully navigated, especially in international collaborations. Additionally, sustained efforts are required to recruit diverse populations, foster trust, and ensure data are used ethically and equitably. The underrepresentation of many groups in large-scale genomic datasets remains a barrier to fully realizing the global impact of precision medicine. Future directions in this field include increasing the integration of WGS with clinical care. As sequencing costs continue to decline, incorporating genomic data into EHRs and healthcare decision-making becomes increasingly feasible. Improvements in machine learning, artificial intelligence, and network-based analyses will further refine genotype–phenotype correlations, predict complex disease outcomes, and highlight novel therapeutic targets. By pushing toward real-time genomics—where sequencing data inform immediate clinical decisions—national genome projects can directly influence patient care, improving outcomes and reducing healthcare disparities.

In conclusion, national genome projects leveraging WGS have expanded the horizons of genetic research, transcending the limitations of earlier genomic approaches. By capturing rare and common variants, coding and noncoding regions, and a spectrum of structural changes, these initiatives are reshaping our understanding of human biology and disease. The future of genomic medicine lies in continued advancements in sequencing technology, scalable analytic frameworks, inclusive research practices, and seamless integration of genomic data into healthcare systems. As these endeavors progress, they promise to deliver innovative solutions that are more equitable, more predictive, and ultimately more beneficial for individuals and communities worldwide.

## Data Availability

No datasets were generated or analysed during the current study.
